# Comments to: A Novel Low-Cost Instrumentation System for Measuring the Water Content and Apparent Electrical Conductivity of Soils, *Sensors*, *15*, 25546–25563

**DOI:** 10.3390/s18061730

**Published:** 2018-05-28

**Authors:** Xavier Chavanne, Alain Bruère, Jean-Pierre Frangi

**Affiliations:** 1 Institut de Physique du Globe de Paris, University Paris Diderot, Sorbonne Paris Cité, UMR 7154 CNRS, Case Courrier 7011, F75205 Paris CEDEX 13, France; frangi@ipgp.fr; 2 CAPAAB, 4 Mail des Houssières, 92290 Chatenay Malabry, France; bruere.alain92@gmail.com

**Keywords:** soil moisture and salinity, permittivity measurement, electrode admittance, self-balanced bridge, Ohm’s law

## Abstract

The article comments on claims made by Rêgo et al. about the sensor they developed to determine soil water content and its salinity via the admittance measurement of electrodes embedded in the soil. Their sensor is not based on a self-balanced bridge, as stated, but on a more common technique relying on Ohm’s law. A bridge is a zero method of measurement which can provide direct voltages proportional to soil permittivity and conductivity with a high resolution. Thanks to modern electronics the method can be adapted for fast and continuous monitoring in a remote site. Because of this confusion about the different measurement techniques among available admittance or capacitance sensors, we give a succinct review of them and indicate how they compare to the two techniques under discussion. We also question the ability of Rêgo et al.’s current sensor to determine both soil water content and salinity due first to instrument biases and then to the soil complexity as a dielectric medium. In particular, the choice of sensor frequencies is crucial in the two steps. In addition, the procedure to determine and account for temperature influences on readings is not presented clearly enough. It is important to distinguish between the effect resulting from electronics sensitivity, and those that are soil-specific. The comment does not invalidate the design of the sensor, but indicates points, especially parasitic contributions, which must be dealt with to avoid major errors.

## 1. Introduction

An operating sensor always presents an output. The question is how it is related to the intended result, here soil water content, $\theta_{v}$, and water salinity, $\sigma_{ion}$. The output can be completely dominated by instrument biases and the goal is unattainable. In addition, a sensor is mostly based on the determination of a convenient but intermediate quantity, here soil relative electric permittivity $\varepsilon_{r}$ and its conjugate soil bulk electric conductivity σ. It is sensitive to our final variable, but work must be done to make the conversion. Of equal importance in science and technique is the correct designation of measurement principles. All of these fundamental considerations are the underlying points of the present comment.

The article [1] describes a novel instrumentation system and its calibration procedure to determine $\theta_{v}$ and $\sigma_{ion}$. As outlined by the authors, among industrial activities, irrigation in agriculture represents the main usage of drinking water, which is increasing with the growth of the population and its well-being. Any means to manage more efficiently this usage, such as cheap but still accurate sensors to monitor continuously and in real time soil water content, $\theta_{v}$, would alleviate the situation. As water salinity in soil, $\sigma_{ion}$, is also an important parameter of crop productivity, its determination is of equal interest. Other applications for which such sensors would be useful should not be forgotten, such as hydrology studies in catchment area [2] or process control (e.g., concrete hardening, compost maturation, moisture in silos...). The sensor determines the intermediate quantities $\varepsilon_{r}$ and σ by measurements of the capacitance and conductance of electrodes inserted in soil. However, some points on the article do not appear clear and others, albeit crucial for the sensor, are not addressed at all. They are the object of this comment. They deal with the correct designation of the capacitance sensor that the authors Rêgo Segundo et al. develop relative to our own work [3,4,5]: the influence of sensor operating frequencies on sensor intended output, and that of the temperature.

As far as the first point is concerned, we contest the authors’ claim that their sensor is an auto-balancing bridge (or self-balanced bridge, as also mentioned), since it is instead based on Ohm’s law, like other sensors [3,6,7]. None of these references claim a sensor based on a self-balanced bridge, although each shares the main feature of the sensor described in [1], at least for the latter two. Partly responsible for this misconception is the impedance measurement handbook of Agilent Technologies, now Keysight Technologies (Santa Rosa, CA, USA), the authors followed too closely (either from the 2003 edition [8] or from the latest one [9]; older editions are also mentioned). It should be noted that the successive editions of the book have fundamentally not evolved since at least 2003 in spite of instrumentation progress. We also observe that in general the literature before 1990 did follow the correct definitions (for instance [10,11]).

Both measurement techniques rely on the contrast presented by soil apparent relative permittivity $\varepsilon_{r}$ (absolute dielectric permittivity normalized by vacuum permittivity $\varepsilon_{0}=8.854$ pF·m${}^{-1}$) between dry and wet soils, as do many other sensors. In addition, conductivity $\sigma_{ion}$ due to the presence of free charges in soil water generates a soil apparent conductivity σ. Both $\varepsilon_{r}$ and σ are tied together with the complex expression $\varepsilon_{r}$ of the permittivity (with $j^{2}=-1$):

(1) $$\varepsilon_{r}=\varepsilon_{r}-j\;\frac{\sigma }{\varepsilon_{0}\;2\pi \;f}\;,$$

where *f* is the frequency of the electromagnetic field produced by sensors to probe the permittivity of a soil, or any porous medium.

**Figure 1:**
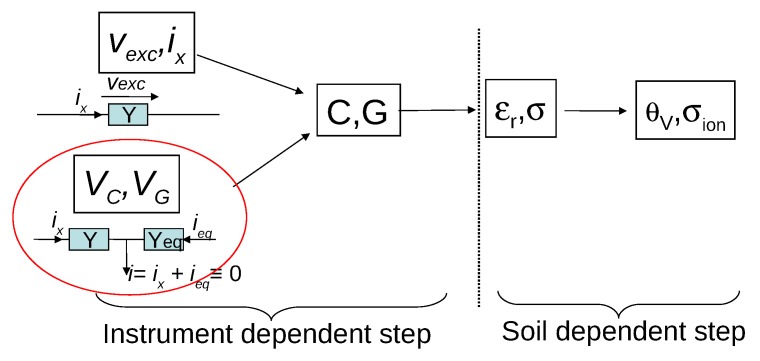
Successive steps of conversion from raw sensor output, direct voltages $V_{G}$ and $V_{C}$ in the case of a self-balanced bridge or alternating current $i_{x}$ and voltage $v_{exc}$ for a method based on Ohm’s law, to quantities of interest, soil water content $\theta_{v}$ and its salinity $\sigma_{ion}$. Quantities *G* and *C* are, respectively, the capacitance and the conductance of the electrodes embedded in the soil, while $\varepsilon_{r}$ and σ are soil apparent electric permittivity and conductivity.

Actually, very few instruments measure the complex quantity $\varepsilon_{r}$ and therefore deduce both $\varepsilon_{r}$ and σ in the same conditions (the same soil sample and frequency). In the next section, for a better overall presentation of the permittivity-based sensors, we will briefly review and classify the various techniques used in available instruments. We then elaborate on the fundamental differences between the two techniques, schematically shown in Figure 1, Ohm’s law-based techniques and actual self-balanced bridge such as the one used in our sensors [4,5]. The technique is derived from the bridge method but modified to be suited for fast and repeated measurements in the field.

Moreover, $\varepsilon_{r}$ and σ are intrinsic quantities of a soil, and therefore independent of the specifications of the instrument that measures them, or should be. To check this point, sensors must be first characterized using references such as passive electronic components or homogeneous fluids of known $\varepsilon_{r}$ and σ. The procedure permits to discriminate between instrument biases and intrinsic electric effects of soil. It is the two-step approach, summarized in Figure 1 in the case of capacitance sensors, now admitted as the standard one [12]. In Section 3 we will show that the frequency *f* adopted in the sensor presented in [1] induces biases in the case of conductive soils, which are not mentioned in the article, even at moderate σ. Besides, the authors must be aware of the difficulty of conversion of $\varepsilon_{r}$ and σ into $\theta_{v}$ and the salinity $\sigma_{ion}$ due to the choice of *f*.

With regard to the temperature, the procedure with known fluids presented in [1] to correct its influence is insufficient and even appears to be useless. Supplementary information given in a later publication by the same authors brings their work closer to a two-step approach as shown in Figure 1. However, it still lacks the consistency of the approach, as we will explain in the same section.

With respect to the two previous articles we wrote about the technique used in our sensors and its performances [4,5], we limit giving new insights or we provide them from a different perspective that is useful for the discussion.

## 2. Distinction between the Various Measurement Techniques of Permittivity-Based Sensors

### 2.1. Overview

The sensor presented in [1], as well as many available soil-moisture sensors, are based on the dependence of soil permittivity on its water content $\theta_{v}$. Permittivity detection is an indirect method to determine $\theta_{v}$, but has the advantage of providing in situ continuous monitoring with autonomous sensors. Indeed, these electromagnetic moisture sensors benefit from progress in information and communication technologies as well as in the manufacturing of integrated circuits (IC), or chips. Operational amplifiers and other active components with high gain-bandwidth product (more than 1 GHz), for the analog part, and micro-controllers with high processing and memory capacities for the digital part make commercial moisture sensors available. However, one constraint for a low cost field system requires that sensor analog output is easy to digitize, such as direct voltages or periodic signals of which frequency is the information to retrieve. On the other hand, permittivity-based sensors must operate at a high frequency to be sensitive to this quantity (and for other reasons developed in Section 3). As a matter of fact, sensor output is rarely related to $\varepsilon_{r}$ in a direct way, but to quantities sensitive to $\varepsilon_{r}$. Hence, the range of techniques used to determine moisture via $\varepsilon_{r}$ is wide. Nevertheless, we can distinguish two important families of sensors (see [5] and references therein for more details):

- Those using the phase velocity or travel time of an electromagnetic wave propagating along a guide in a soil. The velocity varies with the real part of the root square of $\varepsilon_{r}$ (Equation (1)), approximated to $\varepsilon_{r}$ at low conductivity. The most common sensors of this group are the Time Domain Reflectory probes (TDRs) such as CS659 used in the HydroSense system of Campbell Scientific Inc., Logan, UT, USA, or TRIME-TDR of IMKO (Ettlingen, Germany).
- Others using, instead, the amplitude of sensor electromagnetic fields to determine the capacitance (or admittance if they are able to measure simultaneously the conductance part) of electrodes embedded in a soil.

Sensors described in [1] and the ones we develop belong to this last family, which can be cheaper than TDRs due to the lower electronic speed required (signal-rise time higher than few ns, versus less than 10 ps in the case of TDRs). Commercial capacitance sensors usually operate at a fixed frequency *f* lower than 100 MHz. Given an electrode length smaller than 10 cm, their raw output can be thus interpreted thanks to the electric circuit theory, which assumes negligible propagation effect. The sensor electrodes embedded in the soil form a capacitor of which admittance is Y=G+jC2πf with *C* its capacitance and *G* its conductance. *Y* is directly related to the apparent permittivity of the medium $\varepsilon_{r}$ in Equation (1) according to:

(2) $$\varepsilon_{r}=\frac{C}{\varepsilon_{0}\;g}-j\;\frac{G}{\varepsilon_{0}\;g\;2\pi \;f}$$

where *g* is a geometry parameter depending on electrode configuration and dimensions. It is denoted $k_{g}$ in Rêgo Segundo et al.’s article.

However, a further distinction should be made within the family of capacitance sensors, as cheap electronics make a straight measurement of *Y* or even *C* difficult. Capacitance sensors currently available measure either the frequency of an electronic oscillator consisting of an inductor and a capacitor (e.g., the EnviroSCAN and TriSCAN probes marketed by Sentek Pty Ltd. (Stepney, Australia) [13]), or the root mean square of partial-charging cycles of a capacitor (the probes commercialized by Decagon Devices Inc., Pullman, WA, USA) as described in [2]. A hybrid type in the capacitance family uses the propagation and reflection of a sinus wave in a coaxial line ended by the electrodes to determine their capacitance [14,15]. Sensors of Stevens System, Portland, OR, USA (such as HydraProbe), as well as those of Delta_T Devices Ltd., Cambridge, UK (e.g., Theta Probe), are based on this technique, each operating at one frequency (50 and 100 MHz, respectively).

These techniques, if they permit compact and cheap electronics, on the other hand, make their physical modeling difficult. Manufacturers have to resort to empirical conversions to obtain *C* of electrodes, and, consequently, $\varepsilon_{r}$ (corresponding to the first broad step in Figure 1). To obtain a large measurement range and a good accuracy, the conversion requires many empirical parameters. Moreover, they must include in a complex way the dependence with quantities such as the temperature of electronics and soil conductivity σ [13,16]. Sensitivity of the raw output to $\varepsilon_{r}$ is usually not constant over the range of interest. Interpolation between calibration points can produce discrepancies with actual values (see Figure 6 in [5] for Decagon GS3). Conductivity σ can not be measured with the same technique as for $\varepsilon_{r}$, or with poor accuracy (as in the case of HydraProbe).

As a consequence, more direct techniques, which are also able to measure the conductivity, are welcome, provided they result in low-cost and reliable field sensors. In this regard, Ohm’s law-based techniques and actual self-balanced bridge can play an important role.

### 2.2. Ohm’s Law versus Self-Balanced Bridge

We agree with Rêgo Segundo et al. that a certain confusion exists in the literature about the term of self-, or auto-, balancing bridge. Moreover, the sensor designation has no direct importance on its performance. However, it is crucial in science and technique to use precise and unambiguous terms. It is all the more important since we cannot denote two clearly different designs with the same name. To achieve this we do not have to bring a new designation, but we have to name according to the definitions in use within the theory of electric circuits, as seen in textbooks about the field e.g., [17]. As far as the discussion is concerned, there are two basic principles of impedance measurement (see also Figure 1):

- The use of the simple or generalized Ohm’s law, which requires the measurement of voltage and current across the impedance to be determined.
- The Wheatstone, or balanced, bridge, which is a zero method where a reference is adjusted to match the impedance.

#### 2.2.1. Ohm’s Law-Based Techniques

This is the most direct method we can imagine to measure the admittance *Y* of sensor electrodes. The alternating current $i_{x}$ flowing from the electrodes in response to a voltage excitation $v_{exc}$ is related to *Y* as:

(3) $$i_{x}=Y\;v_{exc}=\left(G+j\;C\;2\pi \;f\right)\;v_{exc}.$$

Practical difficulties arise to exploit this relationship. The main one is to succeed in measuring accurately the phase of $i_{x}$ relative to $v_{exc}$, or the in-phase and quadrature components of $i_{x}$, at high frequency (even between 1 and 10 MHz) due to phase errors and inductance effects. The phase of $i_{x}$ coincides to the phase Θ of $Y=Y_{0}\;e^{j\;\Theta }$, which is a useful parameter to assess the capacity of an instrument to determine both *G* and *C*, therefore $\varepsilon_{r}$ and σ, with the same technique (see also Section 3 of [6]). The phase is the inverse of the so-called loss angle and its tangent is:

(4) $$\tan\Theta =\frac{C\omega }{G}=\frac{\varepsilon_{0}\;\varepsilon_{r}\;2\pi \;f}{\sigma }\;.$$

To reach a resolution of $\delta \varepsilon_{r}=\pm 0.1$ for the permittivity at 20 MHz, while having a large apparent conductivity such as σ=100 mS·m${}^{-1}$ (according to Section 4 of [6], the bulk conductivity for clayey soils can exceed σ=100 mS·m${}^{-1}$), the system needs a phase resolution of δΘ≃±0.001 rad or 0.06${}^{\circ }$. The phase itself for a typical permittivity $\varepsilon_{r}=25$ is also low, Θ=0.028 rad or 1.6${}^{\circ }$.

In contrast, converting $i_{x}$ into voltage, or voltage into current, for their subsequent electronic treatment, have become standard electronic operations owing to the progress on IC (precisely thanks to a trans-impedance with one operational amplifier). Our first development of a capacitance sensor relies on this conversion but subsequent operations to determine the phase Θ were not practical or accurate enough for in-field measurements [3].

The difficulty of phase detection did not deter some attempts to use the Ohm based technique. In the 1990s, Hilhorst designed an application specific IC to determine *Y* at f=20 MHz. The circuit contained in particular a remotely actuated switch, a phase shifter from 0 to π/2 and a synchronous detector (i.e., a multiplier of $i_{x}$ and $v_{exc}$ followed by a low-pass filter) to separate the two components of $i_{x}$. Care had to be taken to correct component offsets and phase errors, requiring measurements of on-board references for each piece of data [6].

With progress in electronics, Chang et al. designed a system, also operating at 20 MHz, using a monolithic gain/phase detector (AD8302 of Analog Devices, Norwood, MA, USA) [7]. Its inputs are $i_{x}$, converted into voltage owing to a trans-impedance, and $v_{exc}$. Its output is two direct voltages, one proportional to their phase difference Θ and the other proportional to the logarithm of their amplitude ratio. However, the component linearity is not constant over the range, especially at low phase Θ (phase error of 2${}^{\circ }$ below a phase of few degrees). Data of calibration in the article show an error of δC=15 pf for a reference device with C=33 pf and G=45 mS, equivalent to a phase error δΘ≃±0.04 in rad, or about 2${}^{\circ }$, for Θ=0.09 rad or 0.5${}^{\circ }$. This is too important for our applications.

The system proposed by Rêgo Segundo et al. avoids the difficulty of phase measurement by operating successively at two frequencies, namely 100 kHz and 5 MHz. Each frequency corresponds to the determination of one part of the complex admittance *Y*, respectively *G* and *C*, the other part then being assumed to be negligible. To achieve the separation according to the frequency, the feedback device of a trans-impedance is made of a capacitor $C_{ref}$ and a resistor $1/G_{ref}$ in parallel, acting equally as filters. For each frequency, the amplitudes of $i_{x}$, converted into voltage, and $v_{exc}$ are determined thanks to a rectifier with a low pass filter. Equation (3) thus becomes:

(5) $$|v|=\frac{|Y|}{|Y_{ref}|}\;|v_{exc}|=\sqrt{\frac{G^{2}+{\left(C\;2\pi \;f\right)}^{2}}{G_{ref}^{2}+{\left(C_{ref}\;2\pi \;f\right)}^{2}}}\;v_{exc}.$$

where $|v_{x}|=V_{x}$ denotes the module or amplitude of the alternating voltage $v_{x}$.

If the system is indeed simpler, it does not appear that much different from the one present in the sensor GS3 commercialized by Decagon (Pullman, WA, USA), except that both measurements by the instrument described in [1] share the same electrodes and soil sample. The second objection deals with the ability to separate the two parts of *Y*. To make negligible at f=5 MHz the conductance contribution in the numerator of formula in Equation (5), we must have σ lower than $\sigma =\varepsilon_{r}\;0.28\;$ mS·m${}^{-1}$. It means σ lower than σ=7 mS·m${}^{-1}$ for a typical value of permittivity, $\varepsilon_{r}=25$. It is too low of value relative to those potentially encountered in soils. As *G* is measured independently with a better accuracy than *C*, it is possible to account for it. On the other hand, it still implies a larger uncertainty on *C* determination. Moreover, *G* is determined at a lower frequency and its value may not be the same at f=5 MHz (see the Section 3.1.2 for more details). In the article, the instrument was only tested in low-conductivity soils (σ=10 mS·m${}^{-1}$ at most). During the calibration with saline solutions, the highest value of σ was about σ=80 mS·m${}^{-1}$, which makes tanΘ too low even for $\varepsilon_{r}=80$. It probably explains the dispersion observed in the adjustment curve of the permittivity data for $\varepsilon_{r}=80$. Our final remark concerns the sensor-field frequency; the use of different frequencies between the two measurements and their low values will make the step of conversion of $\varepsilon_{r}$ into soil water content and its salinity more delicate, as developed in Section 3.

We now present a true self-balanced bridge able to measure simultaneously both parts of *Y* at the same frequency and with the output in the form of direct voltages easy to digitize.

#### 2.2.2. Self-Balanced Bridge

Concerning our self-balanced bridge, more details are given in [5] and references therein. Only the principle and the specific features of the bridge are described here. A very first prototype was proposed and developed in the late 1980s to measure continuously the distance between the casement and the moving blades of a gas turbine in operation. The capacitance was the sole quantity measured and the bridge frequency was 100 kHz, increased later to 1.2 MHz [18,19]. The technique has been progressively improved and modified to now measure both *C* and *G* at a frequency as high as 32 MHz.

The basic design is derived from the Wheatstone bridge. The latter consists of a closed loop of four branches, each with a resistor or an admittance. One branch contains the admittance to be determined and another one, adjacent, an adjustable admittance $Y_{eq}$. At two opposite points of the circuit, a fixed supply voltage is applied. $Y_{eq}$ is then modified until the bridge is balanced. At equilibrium, the voltage between the two other opposite points of the circuit—one of which is between the admittances *Y* and $Y_{eq}$—is null, or no current is flowing. $Y_{eq}$ is then proportional to *Y* with a fixed coefficient. This is a zero method of measurement in which the Ohm’s law does not play a direct role. The accuracy results from the uncertainty on the reference $Y_{eq}$ and the ability to measure a null current.

**Figure 2:**
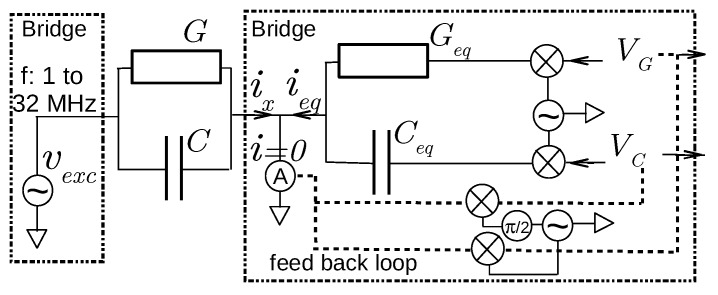
Schematic diagram of a self-balanced bridge. The current $i_{x}$ flowing through the admittance to be measured, Y=G+jC2πf, under the voltage $v_{exc}$ of an on-board oscillator at frequency *f*, is balanced by the current $i_{eq}$ generated by the bridge. $i_{eq}$ is adjusted to $i_{x}$ owing to the direct voltages $V_{G}$ and $V_{C}$ provided by a feedback loop. At equilibrium, they are proportional to $i_{x}$ and therefore *Y*. Synchronous detectors and modulators permit the conversion between alternating and direct voltages using $v_{exc}$ as the alternating reference. Conductance $G_{eq}$ and capacitance $C_{eq}$ give bridge sensitivity and are fixed by passive components.

Obviously, the Wheatstone bridge is not convenient for a field sensor with continuous monitoring. Instead of adjusting the admittance $Y_{eq}$, the supply voltage applied on $Y_{eq}$ is varied while the voltage on *Y*—the voltage $v_{osc}$ of an on-board oscillator at frequency *f*—is kept constant. With this configuration, only the branches of $Y_{eq}$ and *Y* are necessary. A feedback loop from the connecting point between the two branches provides the adjustment to the variable supply voltage in order to cancel the current $i_{x}+i_{eq}$ flowing from this point (see Figure 2). Modern amplifiers provide a null detector with less than 50 pA of uncertainty (or less than 10 nV at the trans-impedance output). The adjustment parameters, which represent the bridge output, are the direct voltages $V_{G}$ and $V_{C}$ obtained from synchronous detectors. The feedback loop and the $Y_{eq}$ part actually contain two parallel branches, one dealing with the in-phase part relative to $v_{exc}$ and the other with the quadrature part. The bridge is thus able to measure simultaneously *G* and *C* thanks to $V_{G}$ and $V_{C}$ according to:

(6) $$\left\{\begin{array}{c} G=G_{eq}\;V_{G},\\ C=C_{eq}\;V_{C}. \end{array}\right.$$

Parameters $G_{eq}$ and $C_{eq}$ give bridge sensitivity and are fixed by passive components. Their values are chosen according to the intended application (in particular low or high expected conductivity). The correction of the phase error of each branch is carried out once with few references.

In addition to bridge phase errors, parasitic impedances between bridge and electrodes must be accounted for, as for any capacitance sensor. The two main ones are the lead and electrode inductances. Their contributions to the signal arise at large σ and *f*. We deal with them owing to an electric model with two parameters, one being fixed in the case of electrodes of known geometry and the other minimized by construction [5]. These effects are rarely mentioned in spite of their importance (except in Section 3 of [6]; however, a rudimentary model is used in that case). They are implicitly, and very approximately, taken into account in the empirical relations used to derive the permittivity from sensor raw signal, thanks to a dependence with the conductivity σ.

Figure 5 in the reference [5] shows, after a few adjustments, the performance of a bridge operating at f=20 MHz to measure $\varepsilon_{r}$ and σ in air and saline solutions. The water permittivity of a solution at about σ=500 mS·m${}^{-1}$ is determined with an uncertainty of $\delta \varepsilon_{r}=\pm 0.5$. It corresponds to a phase resolution of about δΘ≃±0.001 rad or 0.06${}^{\circ }$.

Our instrument is thus entirely based on the Wheatstone bridge and possesses indeed its high resolution (or dynamic, requiring a 16 bit analog-to-digital converter). Thanks to an analog feedback loop, and contrary to the content in the handbook of Hewlett-Packard/Agilent/Keysight, our bridge allows for a fast—the bridge is balanced in less than 10 ms—and continuous response. In addition, our bridge can measure precisely and simultaneously the two parts of the impedance, that is in the same conditions and with the same frequency. It is thus able to resolve the signal phase with very high resolution. The bridge circuit is still simple enough to be integrated into a low-cost autonomous wireless sensor.

**Figure 3:**
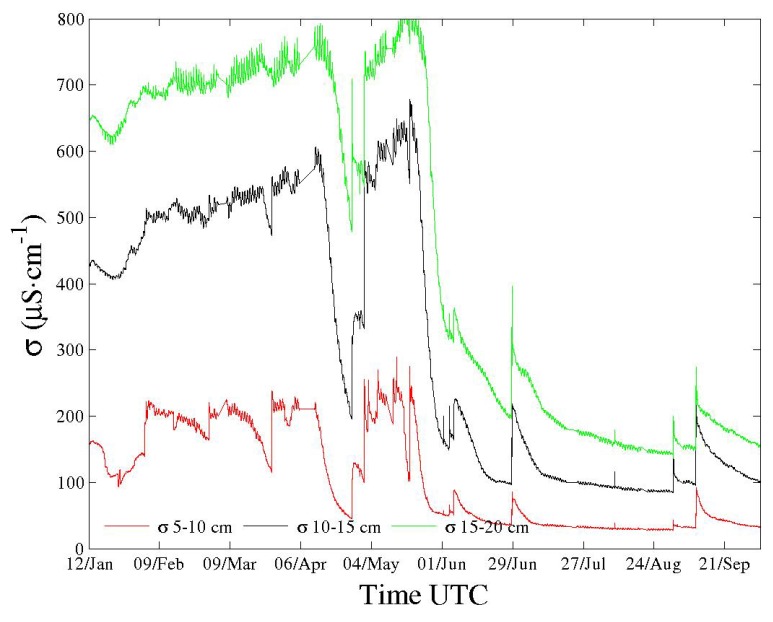
Time series over nine months of a three-channel sensor located in a remote catchment of the French Southern Alps—one point every 10 min. Minor interruptions due to power supply. Soil conductivity profile in μS·cm${}^{-1}$.

**Figure 4:**
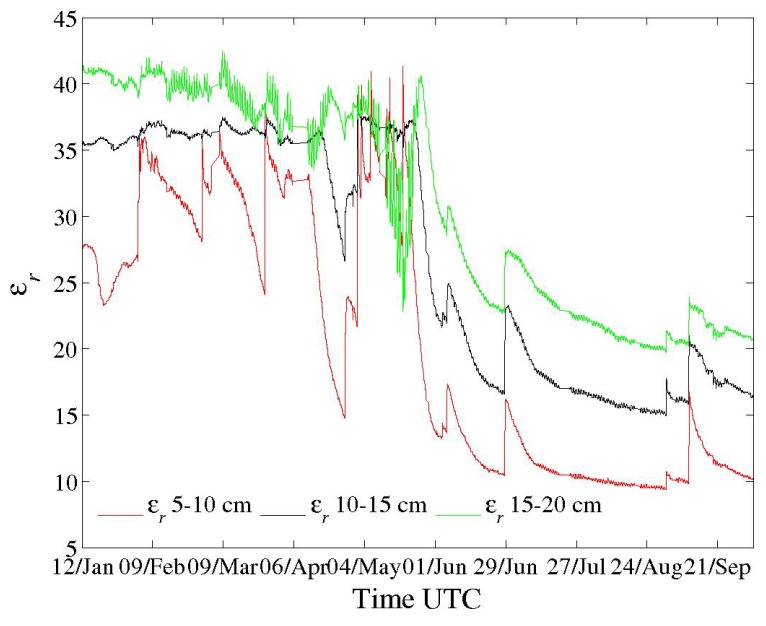
Soil real permittivity profile (see Figure 3).

In terms of field operation, Figure 3 and Figure 4 show the performance of one sensor equipped with a self-balanced bridge. They represent continuous time series over nine months of a three-channel sensor located in a remote catchment of the French Southern Alps [5]. Points are recorded every ten minutes. Figures show soil real permittivity and conductivity profiles. Independently, the sensor provides soil temperature profile using thermometers embedded in electrodes for better thermal contact. All electronics are placed in a watertight box about 18 cm long to 10 cm large. Minor measurement interruptions occurred due to power supply problems and tests, as the corresponding sensor is a first prototype. New sensors dispose of Li-ion batteries within the same housing as the electronics for autonomous operations (about 45 Wh for more than four months). Overall, sensor appearance is similar to the one presented in [1].

#### 2.2.3. Use of High-Gain-Bandwidth-Product Amplifiers in Both Techniques

The idea of a self-balanced or balancing bridge is not new, as noted by Rêgo Segundo et al. For instance, two articles from 1973 describe electric circuits of which principle is close to ours, i.e., they are based on the Wheatstone bridge and use a feedback loop with an integrator to achieve bridge balancing [10,11]. Their circuits were thus not based on Ohm’s law. However, to circumvent the limitation of the components available at that time, they measured only a resistance and/or operated at a low frequency, in the two cases around 25 Hz. Thanks to progress on electronics such as amplifiers with a high gain-bandwidth product, our bridge can operate at more than 20 MHz. This allows an accurate measurement of the capacitance and a less ambiguous interpretation of the permittivity as seen in Section 3.

Both techniques under discussion can measure only a resistance or, with more sophistication, a complex impedance, that is its two parts, a resistance and a capacitance. This level of capability is not a relevant point here. Neither is the use of a trans-impedance, or current-to-voltage converter. The trans-impedance is based on the operational amplifier, of which characteristics are negligible input currents and very high open-loop gain. Hence, if its positive input is grounded, the inverting input is considered at very low voltage, and is commonly called “virtual ground”. Consequently, a virtual ground is a classical technique in modern analog circuits.

**Figure 5:**
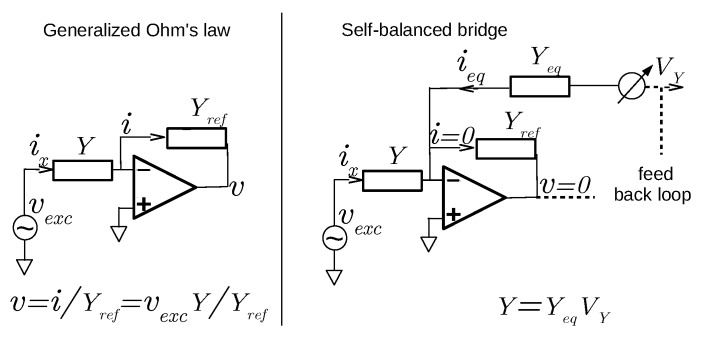
Two techniques in alternating current to measure an unknown admittance *Y* with a trans-impedance—or current-to-voltage converter—in the circuit input: one based on Ohm’s law (left hand part), the other based on the balanced bridge (right hand part). When the bridge is balanced, trans-impedance output *v* is zero (as $i_{x}\equiv -i_{eq}$). The automatic adjustment to reach this state, in form of a direct voltage $V_{Y}$, is used as the sensor signal. In the Ohm’s law case, *v* is one of the sensor signals, along with the excitation $v_{exc}$.

Figure 5 shows trans-impedance use in both measurement techniques. The input of the null detector of our bridge, as described in the articles [4,5], is a trans-impedance used to convert the sum of the alternating currents from both admittances into a voltage for further analog operations. When the bridge is balanced, the sum becomes null and the trans-impedance output *v* is zero. The self adjustment to reach this state, in the form of a direct voltage $V_{Y}$, is used as a sensor signal. In the Ohm’s law case, the output *v* is one of sensor signal, along with the excitation $v_{exc}$.

## 3. Instrument Bias and Soil-Specific Effects

Polarization of a wet soil under an alternating field produces much more phenomena than only the relaxation of the dipoles of its water molecules. In particular, various relaxation phenomena take place over a broad range of frequencies, including those used in permittivity based sensors (electric double layer around grains, Maxwell–Wagner effect, bound water relaxation; see Section 2 of [6] for more details). These effects can make the conversion from $\varepsilon_{r}$ and σ into $\theta_{v}$ and $\sigma_{ion}$ complex, which requires a dielectric model of the soil—that is of an unsaturated porous medium.

In addition, the determination of $\varepsilon_{r}$ and σ from the raw signal can be affected by instrument bias. Both frequency and temperature play a role in the two steps of signal treatment as shown in Figure 1. The difficulty is to separate the two steps, which is possible with the knowledge of the different biases and their characteristics, and thorough calibrations.

### 3.1. Frequency Role

#### 3.1.1. Instrument Bias

We identify three potential instrument biases for which the frequency plays a role, either being too large or too low. As we will see, the soil conductivity is also an important factor, the bias increases with it.

First, we have already seen a bias in Section 2.2.1, with the inability to measure the actual permittivity or electrode capacitance $C=g\;\varepsilon_{r}\;\varepsilon_{0}$ at f=5 MHz even with a moderate conductance G=gσ.

To avoid this effect we can increase the frequency to measure $\varepsilon_{r}$. On the other hand, at frequency higher than 1 MHz, wire and electrode inductances must be taken into account. This effect is also proportional to the length of the wires or the electrodes. In the case of the sensor under discussion, with a distance between the electrodes and electronic circuits higher than 1 m (as in [20]), the total wire inductance can amount to 2 μH, equivalent at f=5 MHz to an important reactance of about 60 Ω, in series with the electrode admittance. With a soil conductivity of about σ=20 mS·m${}^{-1}$ the reactance produces a parasitic contribution to permittivity of about −10 (from Formulae (5) and (18) in [4], using a factor *g* of about 0.15 m). In the extreme case, instead of measuring the permittivity at f=5 MHz, the sensor only detects the conductivity. However, the effect can be compensated and/or accounted for by physical models, as we did for our sensors [5]. Have the authors provided some means in sensor design to prevent this effect?

Measurement at low frequency is not exempt of bias such as the one induced by electrode polarization, as noted by Rêgo Segundo et al. With respect to it, rather than the too succinct and even misleading sentence from Friedman [21], we prefer to refer to the general review on experimental methods and results by Chelidze et al. [22], in particular this extract: “The main difficulty at low frequencies is avoiding electrode effects. A two-electrode cell containing a sample can be considered as two impedances in series which have to be separated: that of the sample and that of the electrode. The effectiveness of separation methods depends on time constants and magnitudes of these impedances. It is obvious that if the frequency responses of the electrode and the sample overlap and if the electrode impedance is a dominant one, the sample impedance is a small part of the total impedance and hence cannot be measured accurately”. The bias depends not only on the frequency but also on values of the surface impedance of the electrodes, $Z_{S}$, and on medium conductivity. Very good electrodes can be obtained in laboratory to reach low frequencies, as described in the article. In the case of our stainless steel electrodes, we performed some measurements with aqueous solutions at various conductivities using the LCR-meter U1733C Agilent to span a frequency range from 100 Hz to 100 kHz, in addition to values provided by our sensors from 1 MHz to 32 MHz (unpublished). With our notation we modeled the resulting admittance $Y_{x}$ as:

(7) $$Y_{x}≃Y\;\frac{G_{S}+j\;C_{S}\;2\pi \;f}{G_{S}+G+j\;C_{S}\;2\pi \;f}.$$

where $C_{S}$ and $G_{S}$ are the capacitance and conductance parts of $Z_{S}$, respectively, such as $Z_{S}=1/\left(G_{S}+j\;C_{S}\;2\pi \;f\right)$. Y=G+jC2πf is the electrode admittance without this effect. The adjustment of our measurement series with Equation (7) gives $C_{S}=5.5\;\mu$F and $G_{S}=0.036\;$ mS. Given the expected range of *Y*, electrode polarization has a negligible effect at 100 kHz, but has an increasing impact at and below 10 kHz for large medium conductivity. It is more pronounced for permittivity than for conductivity. In the case of Rêgo Segundo et al.’s sensor, operating with stainless steel electrodes, like for our sensor, it may be equally the case. Therefore, as far as polarization is concerned, their choice of frequencies seems judicious.

In summary, the authors have to evaluate the behavior of the system relative to all these biases, and possibly modify the choice of frequency, which can be achieved easily with their sensor design.

#### 3.1.2. Soil-Specific Effects

As far as the second step of signal conversion is concerned, many relaxation phenomena take place in soils within the frequency range of Rêgo Segundo et al.’s sensor, which may obscure the determination of the water content (among an abundant literature on the topic see for instance [6,23]). Besides, it is not possible to claim that the apparent conductivity measured by the sensor is the “quasi-static” electrical conductivity used by Friedman [21], which relies on the pioneer work of Archie in 1942. In particular, electric double layers around grains still have a major effect on both $\varepsilon_{r}$ and σ, decreasing with *f*. Moreover, the sensor operates with two different frequencies for measurements of $\varepsilon_{r}$ and σ, 5 MHz and 100 kHz, respectively. Hence they can not use the simple formula given by Hilhorst to convert the conductivity σ into $\sigma_{ion}$, which requires the measurements of the $\varepsilon_{r}$ and σ at the same frequency [24].

It is possible to operate as the authors did but it requires some dielectric models of soils and a determination of some of their characteristics.

### 3.2. Temperature Influence

Like for sensor frequency, the temperature has an influence on the two steps of signal treatment to obtain the water content $\theta_{v}$ and its salinity $\sigma_{ion}$.

In soils, like other investigators using different sensors, we have observed variations of $\varepsilon_{r}$ and σ with soil temperature *T* over the diurnal cycle (see, for instance, Figure 11 and subsequent ones in [5]), but also over larger periods such as in Figure 3 and Figure 4. Interpretation remains difficult not only because of the dielectric complexity of soils but also since soil is an open system through which water moves under various mechanisms (percolation, drainage, evapotranspiration...). Temperature has an indirect action on these mechanisms. All of these mechanisms deal with soil-specific effects.

In addition, the raw signal of the sensor can be sensitive to the temperature of its electronics, $T_{elc}$, which represents an instrument bias. It must be automatically offset, and/or accounted for by means of a software treatment. Surface-mount resistors and capacitors with low sensitivity to temperature (±25 ppm${/}^{\circ }$C) can provide an efficient way to quantify the sensitivity of electronics in the case of capacitance sensors. Using this procedure, we found for our sensors a variation of signal with $T_{elc}$ lower than ±100 ppm${/}^{\circ }$C, which is small relative to field observations. Moreover, the time series of measurements at different soil depths, such as seen in Figure 11 in [5], show that variations of $\varepsilon_{r}$ and σ over the diurnal cycle is correlated to the soil temperature at the same depth, and not to the temperature of sensor electronics, located above soil surface. They are genuine soil-specific effects.

Let us examine now the procedure followed by Rêgo Segundo et al. They studied the variation of sensor signal with temperature by using NaCl aqueous solutions enclosed in a climate chamber. The salinity σ of each solution was measured thanks to a reference apparatus set with an automatic temperature compensation to obtain the value at 25 ${}^{\circ }$C. The temperature is nearly that of the laboratory outside the climate chamber. On the other hand, the sensor itself measures the solution conductivity without any compensation and, therefore, takes into account the variation of σ with the temperature. The correct procedure would have been to disable the automatic compensation of the reference apparatus. It is also important to note that sensor electronics is outside of the chamber. The relation found between the measured conductivity, $\sigma_{m}$, and the one from the conductivity-meter, $\sigma_{25}$ was (the Formula (12) in Rêgo Segundo et al.’s article [1]):

(8) $$\sigma_{25}=\left(-0.0217\;T+1.5698\right)\;\sigma_{m}\;.$$

Logarithm differentiation of this equation gives the linear coefficient of the observed temperature sensitivity:

(9) $$\frac{d\sigma_{m}}{dT}=\frac{0.0217}{1.027-0.0217\;(T-25)}\;.$$

At 25 ${}^{\circ }$C, the coefficient is about 2.1$\%{/}^{\circ }$C, which is exactly the coefficient of variation of the conductivity of an aqueous solution with temperature around 25 ${}^{\circ }$C [25]. It is equally the coefficient used in conductivity-meters to perform the automatic compensation. The relative change is virtually independent of salt nature, KCl or NaCl, as it results mostly from the decrease of water viscosity with temperature. KCl solutions are usually used to calibrate conductivity-meters. As a confirmation, the authors indicate that their relation is similar to one retrieved from Rhoades et al. [26] (at page 6)—Formula (7) in [1]. However, the latter corresponds to the conductivity variation for aqueous solutions, which is exactly our point. As a result, Equation (8) expresses the dependence of the aqueous solution conductivity on *T* and is close to the expected variation.

The same should be said of the solution permittivity, which is approximately that of pure water, $\varepsilon_{r_{w}}$, within the salinity range of the solutions. The Formula (13) in the Rêgo Segundo et al.’s article, which expresses the deduced dependence on *T*, is not as close to the expected and established variation of $\varepsilon_{r_{w}}$ with *T* [27] as for the conductivity, but with the same sign and in the same order. The discrepancy may result from the difficulty to determine the capacitance of sensor electrodes at f=5 MHz, as suggested in the previous paragraph.

The authors then applied their formulae to correct the readings of $\varepsilon_{r}$ and σ obtained with soil samples using the same procedure as for solutions. It partially cancels the effect of temperature variation, mainly on the soil bulk conductivity σ. This result can be explained by the dependence of σ to the ionic conductivity of pore water. However, as mentioned above, the dependence to temperature is far from straightforward due to the complexity of a soil as a dielectric, relative to an aqueous solution.

Their procedure is at worst useless and at best a means to check the capability of their sensor to determine $\varepsilon_{r}$ and σ. In [1], Rêgo Segundo et al. did not study the signal sensitivity to $T_{elc}$. They did it in a later article [20], and provided a formula to account for it in the value of soil water content $\theta_{v}$. The article is confusing as it does not state clearly the existence of two thermometers (transistor-based LM35), one in the electrode plug and another one in the circuit board, which is located 1 m above the soil. The thermometer in the soil, albeit better described, appears definitively useless. The notation for the temperature, *T* instead of e.g., $T_{elc}$, is also misleading. Finally, the correction should be carried out at the step of conversion of sensor signal into $\varepsilon_{r}$ and σ, as the effect corresponds to an instrument bias. All of these points show that the authors do not distinguish properly between electronic bias (with the use of electronics temperature) and physical effect in soil (with the use of soil temperature). Again this is not marginal. However, the distinction can be achieved with a good experimental procedure. For instance, the bias can be determined with electrodes maintained at constant temperature while the temperature of sensor circuit is varied. After removing the bias, soil-specific effects could be studied thanks to the thermometer inside the plug, which measures approximately the soil temperature at the depth of the electrodes.

## 4. Conclusions

This article has discussed some claims, instrument biases and calibration results about a novel instrumentation for the determination of soil water content, $\theta_{v}$, and water salinity, $\sigma_{ion}$, presented in [1]. The sensor belongs to the vast family of electromagnetic sensors using the contrast of soil relative permittivity $\varepsilon_{r}$ between a dry and a wet one. More accurately, the instrument corresponds to a sensor determining the capacitance or admittance of electrodes embedded in a soil, a feature common to many other sensors. It equally provides soil bulk conductivity σ. Independently of the level of its sophistication, its precise technique has been shown to rely on Ohm’s law and not on a self-balanced bridge as stated. The former relies on the measurement of an alternating current and voltage, whereas the latter is a zero method that gives two direct voltages proportional to the admittance to be measured, as realized in our own sensors. The bridge method provides a much higher resolution both in amplitude and phase. It is not possible to confuse it with a Ohm’s law based technique, in spite of a certain ambiguity in the recent literature.

The last points of the discussion concern the capability of Rêgo Segundo et al.’s sensor to determine $\varepsilon_{r}$ and σ, which should be intrinsic soil quantities, taking into account the influence of sensor operating frequencies and temperature on its raw output. Furthermore, both parameters intervene in the final conversion of $\varepsilon_{r}$ and σ into the variables of interest $\theta_{v}$ and $\sigma_{ion}$ and make it difficult due to soil own dielectric complexity.

As far as the frequency *f* is concerned, due to the specific measurement technique, a frequency of 5 MHz can still be too small to deduce $\varepsilon_{r}$. On the other hand, *f* can be too high as a result of the effect of wire inductance. Both biases have not been addressed in the article in spite of their high probability. They also result from soil conductivity, but even a moderate σ can impact the sensor output. With respect to soil-specific effects, some dielectric relaxations occurring at low frequency, such as those from the electric double layer around soil grains, can still be present at 5 MHz. The conductivity σ, determined at f=100 kHz, can be also impacted.

With regard to the temperature influence, the authors found a dependence from the calibration with aqueous solutions. However, it results from the variation of solution permittivity and conductivity with solution temperature. Due to soil dielectric complexity, its application in the case of soils is hardly justifiable. Besides, the effect of electronics temperature on $\varepsilon_{r}$ and σ, and hence on $\theta_{v}$ and $\sigma_{ion}$, is not properly addressed due to the authors’ apparent confusion between instrument biases and soil-specific effects.

The comments do not basically invalidate the design of Rêgo Segundo et al.’s sensors, but must be taken into account, in particular with regard to possible sensor biases.

We thank both the authors and Sensors’ editors for permitting this constructive discussion about the technique of capacitance-based soil moisture sensors.
